# Deficits in reflexive covert attention following cerebellar injury

**DOI:** 10.3389/fnhum.2015.00428

**Published:** 2015-08-04

**Authors:** Christopher L. Striemer, David Cantelmi, Michael D. Cusimano, James A. Danckert, Tom A. Schweizer

**Affiliations:** ^1^Department of Psychology, MacEwan UniversityEdmonton, AB, Canada; ^2^Neuroscience and Mental Health Institute, University of AlbertaEdmonton, AB, Canada; ^3^Glenrose Rehabilitation HospitalEdmonton, AB, Canada; ^4^Division of Neurosurgery, St. Michael's HospitalToronto, ON, Canada; ^5^Division of Neurosurgery, Faculty of Medicine, University of TorontoToronto, ON, Canada; ^6^Keenan Research Centre, St. Michael's HospitalToronto, ON, Canada; ^7^Department of Psychology, University of WaterlooWaterloo, ON, Canada

**Keywords:** cerebellum, attention, covert attention, eye movements, lateral cerebellum, Crus I, Crus II

## Abstract

Traditionally the cerebellum has been known for its important role in coordinating motor output. Over the past 15 years numerous studies have indicated that the cerebellum plays a role in a variety of cognitive functions including working memory, language, perceptual functions, and emotion. In addition, recent work suggests that regions of the cerebellum involved in eye movements also play a role in controlling covert visual attention. Here we investigated whether regions of the cerebellum that are not strictly tied to the control of eye movements might also contribute to covert attention. To address this question we examined the effects of circumscribed cerebellar lesions on reflexive covert attention in a group of patients (*n* = 11) without any gross motor or oculomotor deficits, and compared their performance to a group of age-matched controls (*n* = 11). Results indicated that the traditional RT advantage for validly cued targets was significantly smaller at the shortest (50 ms) SOA for cerebellar patients compared to controls. Critically, a lesion overlap analysis indicated that this deficit in the rapid deployment of attention was linked to damage in Crus I and Crus II of the lateral cerebellum. Importantly, both cerebellar regions have connections to non-motor regions of the prefrontal and posterior parietal cortices—regions important for controlling visuospatial attention. Together, these data provide converging evidence that both lateral and midline regions of the cerebellum play an important role in the control of reflexive covert visual attention.

## Introduction

The cerebellum was once known only for its role in motor functions such as balance and the coordination of movement (for a historical review see Glickstein et al., 2009); however, evidence from recent anatomical, clinical, and neuroimaging studies has suggested that the cerebellum also plays a role in cognitive, affective, and perceptual functions such as attention, memory, language, emotion, and visual, auditory, and motion perception (for reviews see Schmahmann and Sherman, 1998; Cantelmi et al., 2008; Sacchetti et al., 2009; Stoodley and Schmahmann, 2009a,b; Marvel and Desmond, 2010; Stoodley and Stein, 2011; Baumann et al., 2015). These findings are supported by an extensive closed loop neuronal network linking the cerebellum to areas of the cerebral cortex such as the prefrontal and the posterior parietal cortices (Schmahmann and Pandya, 1997; Clower et al., 2001; Dum and Strick, 2003; Strick et al., 2009; Buckner et al., 2011). Although there is clear evidence for the role of the cerebellum in memory, language, emotion, and perceptual functions (Schmahmann and Sherman, 1998; Cantelmi et al., 2008; Sacchetti et al., 2009; Stoodley and Schmahmann, 2009a,b; Marvel and Desmond, 2010; Stoodley and Stein, 2011; Baumann et al., 2015), the role of the cerebellum in attention remains somewhat controversial (Haarmeier and Thier, 2007). Specifically, whereas early studies indicated that the cerebellum played an important role in both spatial and non-spatial attention (Akshoomoff and Courchesne, 1994; Courchesne et al., 1994; Townsend et al., 1999; Schweizer et al., 2007), subsequent studies failed to replicate many of these earlier findings (Dimitrov et al., 1996; Yamaguchi et al., 1998; Ravizza and Ivry, 2001; Golla et al., 2005). This led to the suggestion that earlier studies in which patients with cerebellar lesions appeared to have attentional deficits may have stemmed from motor impairments (i.e., slowed button presses, slowed eye movements) that masqueraded as attentional impairments (Ravizza and Ivry, 2001; Haarmeier and Thier, 2007).

One important question to consider is what role, if any, the cerebellum might play in attention. Given the cerebellum's role in coordinating motor output, it is conceivable that the cerebellum may help control attention by influencing the motor effectors involved in generating attentional shifts. Specifically, it is well known that attention and eye movements share largely overlapping neural substrates (Corbetta et al., 1998; Colby and Goldberg, 1999; Nobre et al., 2000; Astafiev et al., 2003). This is consistent with the premotor theory of attention which posits that covert shifts of attention (i.e., shifts of attention without moving the eyes) simply represent saccades that are planned, but not executed (Rizzolatti et al., 1987, 1994). Thus, the cerebellum may influence attention through circuits that are also involved in the execution of eye movements.

Given the close link between attention and eye movements it is important to try to separate the cerebellar contributions to these two processes. One experimental paradigm that has been used rather extensively in this regard is the covert orienting of visual attention task developed by Posner and colleagues (Posner et al., 1980, 1984, 1987). In this task participants are asked to covertly attend (i.e., without moving their eyes) to marked locations to the left and right of fixation. At the beginning of a trial a cue is presented (e.g., a peripheral flash or a directional arrow). Following the cue, a target appears either in the cued location (i.e., a validly cued trial) or in the opposite, uncued location (i.e., an invalidly cued trial). Typically, participants are faster to detect the target when it is validly cued compared to when it is invalidly cued (i.e., the cuing effect). Using this paradigm it is also possible to cue participants exogenously (i.e., reflexively) or endogenously (i.e., voluntarily) by manipulating both the type of cue used, as well as the cue-target probability. Specifically, participants can be cued reflexively using abrupt onset peripheral cues that capture the participant's attention. Importantly, these peripheral cues are effective at attracting the participant's attention at short stimulus-onset-asynchronies (SOAs; i.e., 50 ms), even when the cues are non-predictive (i.e., 50% valid). However, at longer SOAs (i.e., >250 ms) inhibition of return (IOR) is observed where participants are faster at detecting invalidly cued targets compared to validly cued targets (Posner et al., 1985; Klein, 2000). In contrast, voluntary attention is typically examined with this task using a predictive central arrow cue (i.e., 80% valid) to indicate where the participant should allocate their attention, coupled with longer SOAs to allow for the generally slower orienting associated with endogenous mechanisms (Posner et al., 1980; Muller and Rabbitt, 1989; see Ristic and Kingstone for an alternative viewpoint, Ristic and Kingstone, 2006).

Previous studies that have examined covert attention in cerebellar patients have produced mixed results. An early study by Townsend et al. (1999) demonstrated that cerebellar patients had deficits in reflexive (i.e., exogenous) attention such that they required additional time to direct their attention toward a peripheral cue. Interestingly, this deficit was correlated with a decreased volume in lobule VI of the cerebellum, a region that is known to be involved in the control of eye movements (Robinson and Fuchs, 2001). However, subsequent studies failed to replicate these findings (Dimitrov et al., 1996; Yamaguchi et al., 1998; Golla et al., 2005). One consistent problem with the studies that failed to find any relationship between cerebellar damage and deficits in covert attention is that they used heterogeneous patient populations. Specifically, each of these studies combined patients with cerebellar damage and patients with diffuse cerebellar degeneration. This implicitly assumes that all areas of the cerebellum should be equally involved in attention; however there is ample evidence for functional specialization within the cerebellum (Stoodley and Schmahmann, 2009b; Glickstein et al., 2011). Thus, if heterogeneous patient populations are used, it may be unlikely that a clear deficit in attention will be identified.

A more recent study by Baier et al. (2010) examined covert attention in patients with circumscribed cerebellar lesions by comparing each patient separately to the overall group. Their results indicated that a small subset of patients (8 of 26) demonstrated clear covert attention deficits following damage to vermal regions of the cerebellum that are known to be involved in the control of eye movements. Furthermore, a recent neuroimaging study in healthy individuals provided converging evidence for these patient findings demonstrating that the same cerebellar region—lobule VI of the oculomotor vermis—was involved in both eye movements and covert attention (Striemer et al., 2015). Both studies confirm the earlier findings of Townsend et al. (1999), that oculomotor structures in the cerebellum are involved in covert shifts of attention.

Although previous work indicates that oculomotor structures in the cerebellum play a role in generating covert shifts of attention, it is unclear whether additional cerebellar structures might also play a role. A number of studies indicate that the cerebellum is interconnected with several non-motor regions of the cerebral cortex (via the cerebellar dentate nucleus) including the dorsal lateral prefrontal cortex (area 46), the frontal eye fields (FEF), and several sub-regions of the posterior parietal cortex (PPC; areas 7b, AIP, MIP, and LIP; Lynch et al., 1994; Clower et al., 2001, 2005; Middleton and Strick, 2001; Dum and Strick, 2003; Strick et al., 2009; Prevosto et al., 2010). Several of these PPC regions, as well as the FEF, are known to play important roles in controlling attention (Colby and Goldberg, 1999; Corbetta and Shulman, 2002; Goldberg et al., 2006; Corbetta et al., 2008). Thus it is plausible that other cerebellar structures not strictly tied to oculomotor control might also play a role in covert attention by acting on these same cortical regions. To further examine this possibility we studied the effects of cerebellar lesions on covert attention performance in patients without gross oculomotor deficits.

## Methods

### Participants

Eleven patients with cerebellar lesions participated in this study (5 female; age range 26–70 years, mean 48.8 years). All patients had focal lesions (6 with left hemisphere lesions, 5 with right hemisphere lesions) restricted to the cerebellum, as determined by post-surgical MRI or CT scan, caused by either the removal of a benign tumor (*n* = 8), or a stroke (*n* = 3). None of the tumor patients were treated with chemo-or radiation therapy. All patients were tested at least 90 days post-surgery (mean 1207 days, median 1095 days). All patients self-reported as being right handed. Patients were excluded from the study if: (1) they were diagnosed with conditions that could cause cognitive impairments, such as global changes in white matter, psychiatric disorders, hydrocephalus, ischemic disease, neurodegenerative disorders, or prior traumatic brain injury; (2) if they were using medications or substances that could affect cognitive functions; (3) standard clinical neurological assessment revealed significant motor impairments, including oculomotor dysfunctions such as saccadic dysmetria, or difficulty with smooth pursuit eye movements. Patient demographics and clinical data are reported in Table 1.

**Table 1:** **Patient demographics and clinical data**.

**Patient**	**Gender**	**Age**	**Education (years)**	**Etiology**	**Time post injury (days)**	**Side of lesion**	**Lesion location**
1	F	55	12	Tumor	1095	R	VIIIA, VIIB, CrII, CrI
2	F	43	12	Tumor	1144	R	VIIIA, VIIB, CrII, CrI
3	M	42	21	Vascular	744	L	VIIA, VIIB, VIIIA, CrI, CrII
4	M	60	8	Tumor	352	L	VIIB, CrI
5	M	51	12	Tumor	1151	L	CrI, CrII, VI
6	F	70	12	Tumor	3864	L	CrI, CrII, VIIB, III, IV, V, VI
7	M	44	12	Tumor	1532	R	VIIIA, VIIIB
8	F	26	15	Tumor	1058	R	CrI, CrII, VIIB
9	F	63	16	Vascular	864	R	I, II, III, IV, V, VI, VIIIA, CrI
10	M	36	14	Tumor	1379	L	VIIIA, VIIB, CrI, CrII
11	M	47	12	Vascular	91	L	VIIIA, VIIB, CrI, CrII, V, VI

Eleven control subjects (7 female; age range 24–73 years, mean 49 years) also participated in this study. Independent sample *t*-tests were used to confirm that the patients and controls were matched with respects to age (*p* = 0.78) and years of education (*p* = 0.10). All participants gave written consent prior to participating in this study. The experimental procedures used were approved by the Research Ethics Board of St. Michael's Hospital (Toronto, ON, Canada) in accordance with the Declaration of Helsinki.

### Setup and procedure

The covert attention task was presented on an IBM compatible Pentium 4 notebook computer with a 15.4” screen running Superlab 2.0 (Cedrus, CA, USA) software. Responses were recorded with an external button press (RB-530; Cedrus, CA, USA). Participants were seated 60 cm from the monitor with their head in a chin-rest. The response pad was aligned to the participant's body midline.

**Figure 1:**
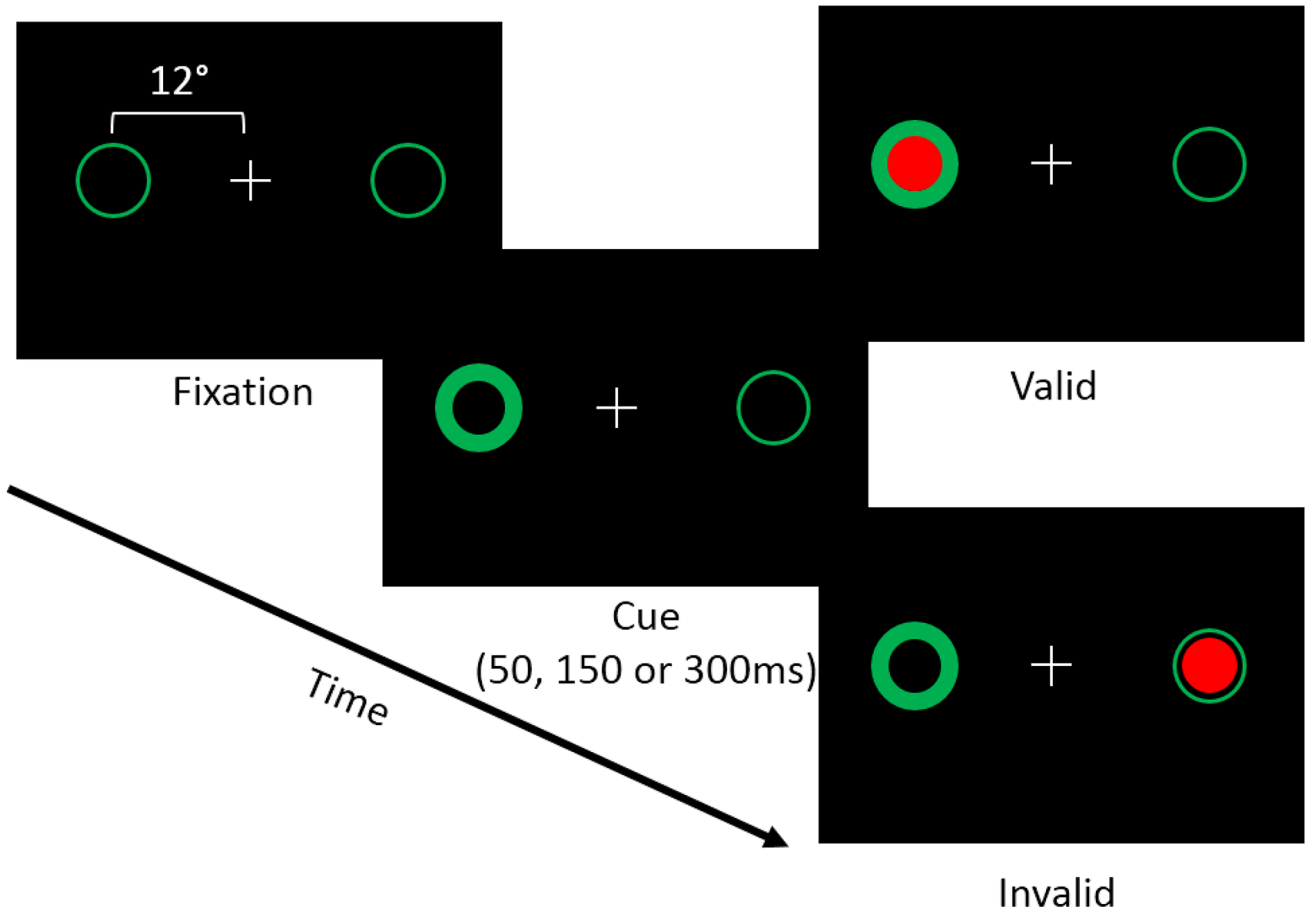
**Depicts the layout of the covert attention task**. Participants were asked to fixate while attending to landmarks (i.e., green circles) located 12° to the left and right of fixation. At the beginning of a trial one of the two landmarks would brighten, reflexively cuing the participant's attention to that location. Following an SOA of 50, 150, or 300 ms a target (i.e., a red circle) would appear either at the cued (i.e., valid) or the uncued (i.e., invalid) location. Participants were asked to respond via a button press when the target appeared on the screen.

Each trial of the covert attention task began with a white central fixation cross presented on a black background. Possible target locations were marked using green circles (subtending 2 degrees of visual angle) located 12° to the left and right of fixation (i.e. peripheral landmarks; Figure 1). At the beginning of a trial one of the green circles brightened, which resulted in a reflexive shift of attention to the cued location. Following the cue, a target appeared either in the same location (i.e., valid trial), or the opposite location (i.e., an invalid trial). Targets consisted of filled red circles that appeared within one of the two peripheral landmarks. Cues were non-predictive (i.e., 50% valid) of target location. In addition, we manipulated the time between the onset of the cue and the target (i.e., the stimulus onset asynchrony; SOA) such that the target appeared either 50, 150, or 300 ms following the onset of the cue. The primary dependent measure was the participant's reaction time (RT) to detect the onset of the target. We chose to use a reflexive covert attention task because previous studies have indicated that cerebellar lesions primarily disrupt reflexive covert attention (Townsend et al., 1999; Baier et al., 2010). Furthermore, a recent fMRI study in healthy individuals demonstrated that the cerebellum was more active in a task that measured reflexive (exogenous) compared to voluntary (endogenous) covert orienting (Striemer et al., 2015).

In addition to validly and invalidly cued targets we also included non-cued trials in which a target would appear on either the left or right side of the screen without any preceding cue. We used these trials to measure simple target detection in the absence of cuing. Finally, we also included “catch trials” in which both cues would brighten simultaneously and no target was presented, and thus, no response was required. These trials were used to prevent anticipatory responses, and to ensure that participants were attending to the task.

Prior to beginning the task, participants were instructed to keep their eyes on the fixation cross at all times and to use their dominant hand to press the button on the response pad as quickly as possible when a target appeared. Participants were reminded to keep their eyes on the fixation cross at approximately 4 min intervals during the task. Prior to the main experiment each participant (both patients and controls) completed 10 practice trials in which their eye movements were directly observed by the experimenter. None of the participants (patients or controls) had any difficulty maintaining fixation during the task. Given that the targets were located 12° in the periphery, this would have made any eye movements to the target easily visible. The covert attention task for the main experiment contained 280 randomized trials that consisted of 20 trials for each cue (valid vs. invalid) by target (left vs. right) by SOA (50, 150, 300) combination (i.e., 240 trials). We also included 20 non-cued trials (10 for each target location) and 20 catch trials.

### Statistical analyses

For each participant we calculated the mean RT for each condition. Reaction times exceeding 2 standard deviations (SD) for the participants' overall mean RT for that condition were eliminated as outliers. In addition, RTs below 150 ms were considered to be anticipatory responses and were removed from further analysis. This accounted for less than 5% of trials in all participants. RT data were then analyzed using a mixed model ANOVA with group (control vs. patient) as the between-subject factor and cue (valid vs. invalid), target (left vs. right), and SOA (50, 150, 300) as within-subject factors. *Post-hoc* tests were carried out when necessary using *t*-tests with a Bonferroni correction.

### Lesion site analysis

Post-acute CT and MRI (T1 via a 1.5T MRI) scans were used to identify areas of cerebellar damage in each patient. Lesion locations were identified in each patient by a neurologist and verified by a neurosurgeon who were not informed of the hypothesis of the study. Lesion locations were then plotted onto anatomical templates consisting of 12–4 mm thick axial cerebellar slices based on the templates first published by Tatu et al. (1996). Lesioned regions of the cerebellum were then identified using the MRI atlas of the human cerebellum (Schmahmann et al., 1999; Schmahmann, 2000).

## Results

### Differences in covert attention performance between patients and controls

Mean reaction time (RT) data for each condition for each patient as well as the control group are presented in Table 2. The results of the ANOVA revealed main effects of cue [*F*_(1, 20)_= 23.99, *p* < 0.001] and SOA [*F*_(2, 40)_ = 3.87, *p* = 0.029] such that participants responded more quickly for validly (431 ms) compared to invalidly cued targets (449 ms), and were slower to respond to targets at the 50 ms SOA (449 ms) compared to the 150 ms SOA [436 ms; *t*_(21)_ = 2.99, *p* = 0.021 corrected]. Interestingly, there was no main effect of group (*p* = 0.56), indicating that, overall, RTs were similar between patients and controls.

**Table 2:** **Mean reaction time (RT) data for controls (*n* = 11) and cerebellar patients (*n* = 11) as a function of side of target (left vs. right), stimulus onset asynchrony (SOA; 50, 150, 300 ms), and cue type (valid, invalid, no cue)**.

	**Left**							**Right**						
	**50**		**150**		**300**			**50**		**150**		**300**		
	**Valid**	**Invalid**	**Valid**	**Invalid**	**Valid**	**Invalid**	**No Cue**	**Valid**	**Invalid**	**Valid**	**Invalid**	**Valid**	**Invalid**	**No Cue**
Controls (*n* = 11) (*SD*)	421 (38)	465 (43)	433 (38)	439 (42)	447 (54)	433 (52)	449 (39)	413 (32)	453 (31)	409 (46)	443 (39)	423 (39)	420 (33)	449 (40)
Patient 1	415	437	368	398	403	403	499	396	434	388	385	405	395	447
Patient 2	626	608	606	609	478	591	604	615	647	609	606	588	586	679
Patient 3	526	546	539	540	495	491	557	563	552	524	537	482	465	572
Patient 4	399	462	404	378	421	454	440	409	409	408	404	445	450	427
Patient 5	444	457	368	415	343	358	499	431	460	365	423	352	399	486
Patient 6	352	353	401	355	380	350	385	338	334	341	354	354	332	371
Patient 7	481	510	454	518	461	533	497	520	497	420	483	502	509	509
Patient 8	419	434	401	401	415	366	439	429	460	400	420	367	364	440
Patient 9	370	433	367	402	371	434	407	384	439	383	429	415	420	404
Patient 10	469	502	480	512	474	533	567	476	513	422	497	473	526	518
Patient 11	379	431	430	419	436	443	419	415	426	417	430	439	420	405
Patient group mean (*SD*)	444 (80)	470 (68)	438 (77)	450 (81)	425 (49)	450 (80)	483 (72)	452 (83)	470 (82)	425 (76)	452 (73)	438 (71)	442 (75)	478 (89)

*Standard deviations are in brackets*.

In addition to the main effects, there was a significant cue × target × SOA interaction [*F*_(2, 40)_= 6.15, *p* = 0.005]. To further examine this three-way interaction we analyzed the cue × target interaction separately at each SOA. This analysis revealed that the cue × target interaction was significant at the 150 ms SOA [*F*_(1, 21)_ = 9.81, *p* = 0.005], but not at the 50 or 300 ms SOAs (*p*'s > 0.36). *Post-hoc* paired samples *t*-tests demonstrated that, at the 150 ms SOA, participants were significantly faster for validly cued targets that appeared in the right visual field (417 ms) compared to the left visual field [436 ms; *t*_(21)_ = 3.61, *p* = 0.004 corrected]. However, there was no significant difference between RTs for invalidly cued targets in the left (444 ms) and right visual fields [447 ms; *t*_(21)_ = 0.59, *p* = 0.56].

**Figure 2:**
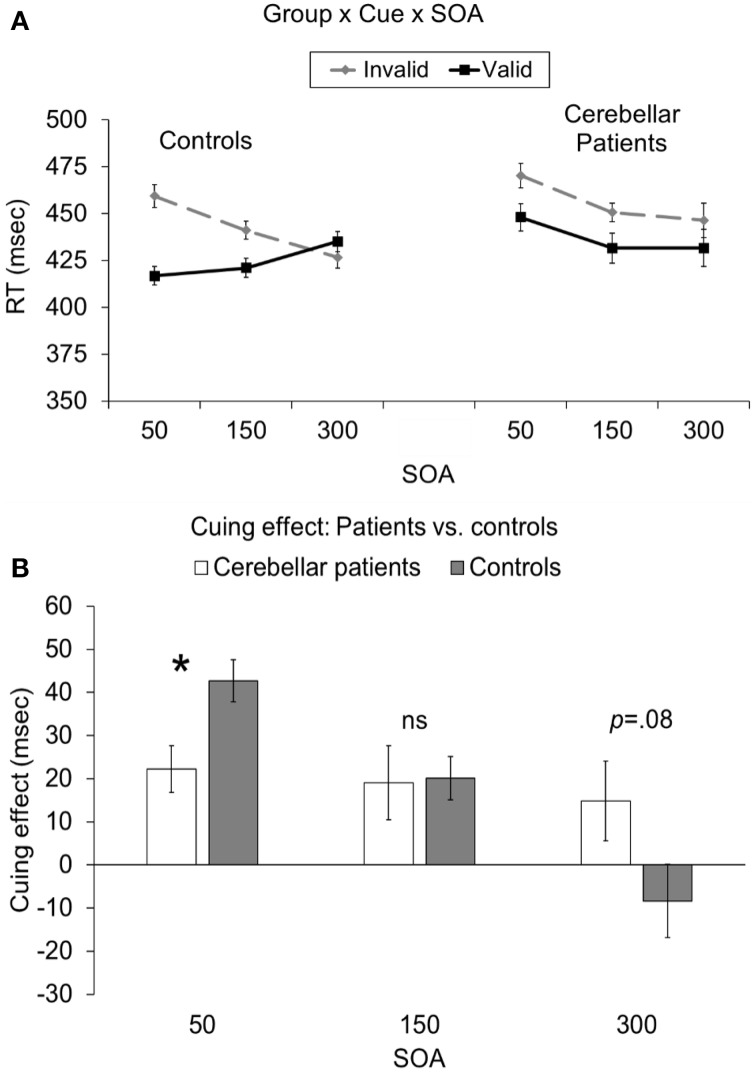
**(A)** Depicts the group (patients vs. controls) × cue (valid vs. invalid) × SOA (50, 150, 300 ms) interaction. **(B)** Depicts the cuing effect (invalid RT—valid RT) as a function of group (patients vs. controls) and SOA (50, 150, 300). In both graphs the error bars represent the within-subject standard error (Loftus and Masson, 1994). Statistically significant differences are denoted by ^*^p < 0.05, corrected.

Critically, there was also a significant cue × SOA × group interaction [*F*_(2, 40)_ = 6.78, *p* = 0.003; Figure 2A]. This interaction indicated that the cuing effect (i.e., invalid-valid) was significantly larger for controls (43 ms) compared to cerebellar patients (22 ms) at the 50 ms SOA [*t*_(20)_ = 2.81, *p* = 0.033 corrected; Figure 2B]. However, there was no significant difference in the cuing effect between the two groups at the 150 ms [controls = 19 ms, patients = 20 ms; *t*_(20)_ = 0.1, *p* = 0.92] or the 300 ms SOAs [controls = 15 ms, patients = −8 ms; *t*_(20)_ = 1.84, *p* = 0.08, 0.24 corrected]. In order to further analyze this effect we directly compared the RTs for valid and invalid trials at the 50 ms SOA in the patients to those of controls using independent samples *t*-tests assuming unequal variance. Although the RTs for validly cued targets in the patients (448 ms) appeared to be slower than controls (417 ms), this difference was not statistically reliable [*t*_(13)_ = 1.18, *p* = 0.25]. In addition there was no significant difference for invalidly cued RTs between patients (470 ms) and controls [460 ms; *t*_(13)_ = 0.44, *p* = 0.66]. To summarize, although the effects of cerebellar lesions were not exclusive to either validly or invalidly cued trials at the 50 ms SOA, the *overall pattern* (i.e., invalid-valid) of reaction times at this early SOA was significantly different between the groups. The reduced cuing effect in the cerebellar patients at the 50 ms SOA was remarkably consistent such that 10/11 patients demonstrated a cuing effect that was smaller than the mean of the controls. This was confirmed using a Wilcoxon Signed Rank Test (*p* < 0.033).

In a follow-up analysis we examined whether time post-injury was related to our results. To analyze this we correlated the magnitude of the cuing effect at the 50 ms SOA (i.e., invalid-valid) with the number of days post-injury (Table 1). This analysis revealed no significant relationship between the cuing effect at the 50 ms SOA and time post-injury [*r*_(11)_ = −0.51, *p* = 0.11]^1^. In addition, we also examined whether the reduced cuing effect observed in the cerebellar patients was related to the side (i.e., left vs. right) or relative size of the lesion. When we compared the magnitude of the cuing effect at the 50 ms SOA for patients with left (*n* = 6, *P*'s 3–6, 10, 11; see Supplemental Figures) or right (*P*'s 1, 2, 7–9) sided lesions, we found no significant differences in performance [left (20 ms) vs. right (24 ms); *t*_(9)_ = 0.36, *p* = 0.72]. In terms of lesion size, we categorized patients as having a “small” (*n* = 7; *P*'s 2–4, 6–8, 10) or “large” (*n* = 4; *P*'s 1, 5, 9, 11) lesion based on their individual lesion maps (see Supplemental Figures). The results of this analysis revealed a non-significant trend toward a smaller cuing effect in patients with small (15 ms) compared to large (35 ms) lesions [*t*_(9)_ = 2.14, *p* = 0.061]. It is important to note that the small sample sizes for our patient sub-groups suggest these analyses should be interpreted with caution.

Finally, we analyzed the non-cued trials (Table 2) using a mixed ANOVA with side of target (left vs. right) as a within-subject factor, and group (patients vs. controls) as a between subject factor. There were no significant main effects or interactions indicating that RTs for non-cued trials did not differ between the groups.

### Lesion-site analysis

Visual inspection of the lesions in the cerebellar patients indicated that the areas of greatest overlap (darker gray) were centered largely in the regions of Crus I and Crus II of the lateral cerebellum (Figure 3). Lesion maps of individual patients are provided as Supplementary Figures 1–11.

**Figure 3:**
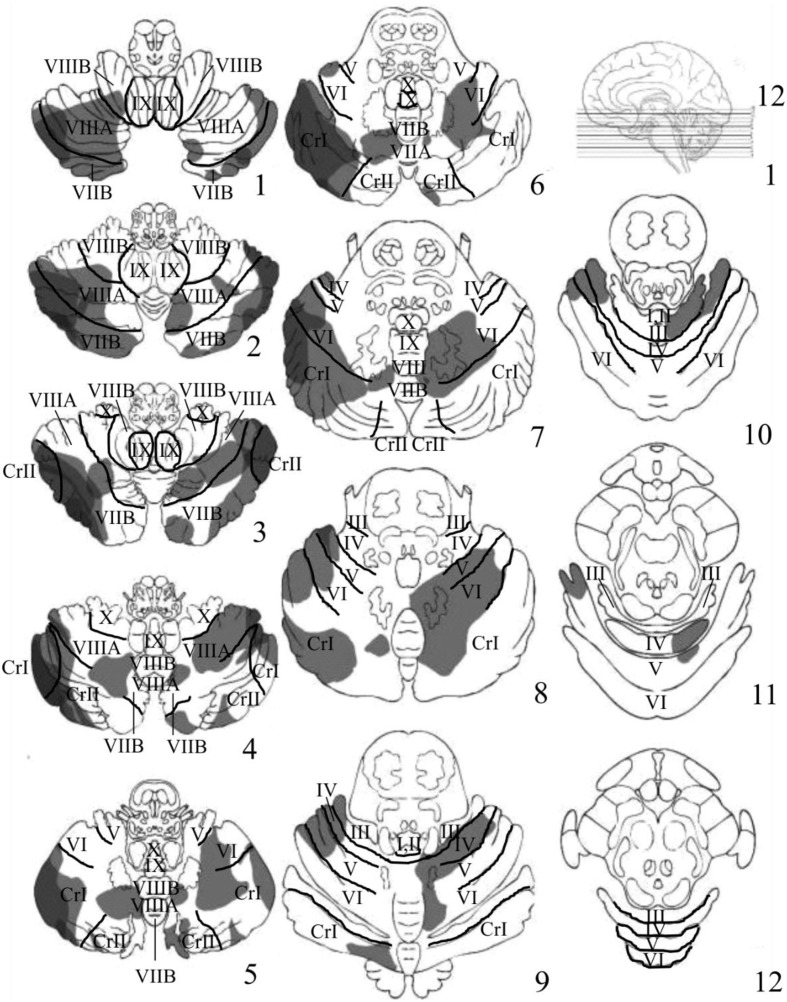
**Depicts the results of the lesion overlay analysis for the cerebellar patients (*n* = 11)**. Lesioned areas in each patient were manually traced onto a series of 12-4 mm thick axial cerebellar slices based on the templates first developed by Tatu et al. (1996). Structural labels and anatomical boundaries were determined by comparing sections of the cerebellar template with horizontal MRI and histology sections from the MRI Atlas of the Human Cerebellum (Schmahmann, 2000). Slice 1 starts at the most inferior portion of the cerebellum and moves upward in 4 mm increments toward more superior portions of the cerebellum. Gray portions represent the area of the patient's lesions. Darker gray regions represent areas of significant overlap between patients. The regions of greatest overlap were in Crus I and Crus II of the lateral cerebellum.

## Discussion

Traditionally the cerebellum has been known for its important role in coordinating motor output (Glickstein et al., 2009, 2011); However, a number of studies over the past 20 years have demonstrated that the cerebellum appears to be involved in a variety of higher level cognitive functions such as working memory, language, emotion, and perceptual functions (Sacchetti et al., 2009; Stoodley and Schmahmann, 2009a,b; Marvel and Desmond, 2010; Baumann et al., 2015). In addition, a growing body of evidence suggests that regions of the cerebellum involved in oculomotor control are also involved in covert visual attention (Townsend et al., 1999; Baier et al., 2010; Striemer et al., 2015). We were interested in determining whether cerebellar structures not traditionally associated with oculomotor control might also play an important role in controlling covert visual attention. To investigate this we examined reflexive covert attention performance in a group of patients with cerebellar lesions (*n* = 11) who did not display any gross motor or oculomotor deficits during a standard neurological examination, and compared them to a group of healthy age-matched controls (*n* = 11).

The results indicated that the RT advantage for validly compared to invalidly cued targets was dramatically reduced in our patients at the earliest SOA (i.e., the cue effect was roughly half that of controls; Figure 2). Importantly, overall RTs were equivalent across the two groups suggesting that the difference observed at the earliest SOA cannot simply be due to some generalized slowing of performance.

It is not entirely clear why our cerebellar group was not significantly slower overall than the control group. Many previous studies that have measured RT in cerebellar patients and have noted a general slowing of RTs have primarily examined patients with widespread cerebellar degeneration rather than patients with circumscribed lesions as tested here (e.g., Yamaguchi et al., 1998). However, other studies that have examined patients with circumscribed lesions have noted that many patients have normal RTs compared to controls. For example, in a study by Baier et al. (2010) 18 of 26 patients had RTs that were within the range of healthy controls. Time post lesion might also be a contributing factor. Specifically, on average our patients were 3.3 years (mean = 1207 days; Table 1) post injury making it likely that some degree of recovery of motor functions had occurred. Furthermore, we excluded patients from our study that had gross motor and oculomotor impairments (see Methods). The cerebellar regions most directly linked with the skeletomotor system are located more medially in the cerebellum in the anterior lobe, and lobules VIII and VIIb (Glickstein et al., 2011). Given that we excluded patients with gross motor impairments this would have excluded patients with more medial lesions and thus reduced the chances of us finding an overall slowing of RTs. The critical point to keep in mind, however, is that even though there was no overall slowing of RTs for patients compared to controls, the *pattern* of RTs (i.e., invalid-valid) differed significantly between patients and controls. Thus, the lack of a generalized impairment (that would have been evidenced in a slowing of overall RTs) suggests that our data cannot simply be dismissed as being due to non-specific motor output deficits as others have postulated (Haarmeier and Thier, 2007).

In addition, it is also unclear why deficits in covert attention in the cerebellar group were most prominent at the 50 ms SOA; However, it could be the case that the speed with which patients can shift attention to a cued location is slowed following cerebellar injury and that, with more time, their performance can “catch up” (i.e., their cuing effect was normal at the 150 ms SOA). This is consistent with previous work by Townsend et al. (1999) in which they were able to demonstrate slowed covert shifts of attention at shorter SOAs and normal covert attention at longer SOAs.

Another interesting finding was a trend toward a decreased inhibition of return (IOR; i.e., faster RTs for invalid compared to valid trials) at the 300 ms SOA in patients compared to controls (*p* = 0.08, uncorrected). Although these data are suggestive of a cerebellar contribution to IOR, future studies should use longer SOAs (i.e., >300 ms) to further investigate this possibility. Finally, the inspection of the lesions in the cerebellar group indicated that the areas of greatest overlap were in Crus I and Crus II of the lateral cerebellum (Figure 3).

Our results are consistent with previous work demonstrating that the cerebellum plays an important role in controlling reflexive covert attention (Townsend et al., 1999; Baier et al., 2010; Striemer et al., 2015). Any previous work that has failed to replicate these findings either did not examine reflexive attention, or examined heterogeneous groups of patients, making any observable link between cerebellar damage and covert attention unlikely (Dimitrov et al., 1996; Yamaguchi et al., 1998; Golla et al., 2005). Interestingly, the reduced cuing effect in cerebellar patients at the 50 ms SOA suggests that they are not able to rapidly orient reflexive attention; however, they are able to orient their attention toward cues at longer SOAs.

Inspection of lesions indicated prominent overlap in Crus I and Crus II of the lateral cerebellum. Significant activation in these regions has been observed in previous neuroimaging studies examining both covert visual attention (Lepsien and Pollmann, 2002), non-spatial attention shifting (Allen et al., 1997), eye movements (Ron and Robinson, 1973; Straube et al., 1997; Dieterich et al., 2000), and numerous other, higher level cognitive tasks (Stoodley and Schmahmann, 2009b). Interestingly, Crus I and Crus II are known to send inputs to the dentate nucleus of the cerebellum, which in turn projects to the PPC and FEF (Lynch et al., 1994; Voogd, 2003; Strick et al., 2009; Prevosto et al., 2010; Glickstein et al., 2011). A recent fMRI study in humans examining intrinsic functional connectivity between the cerebellum and the cerebral cortex observed that Crus I and Crus II have a great deal of functional connectivity with non-motor regions within the dorsal and ventral prefrontal cortex, as well as the PPC (Buckner et al., 2011). Previous neuroimaging studies have shown that these same regions of prefrontal and posterior parietal cortex are important for the control of visuospatial attention (Corbetta et al., 1998, 2000, 2008; Nobre et al., 2000; Corbetta and Shulman, 2002; Lepsien and Pollmann, 2002; Astafiev et al., 2003; Striemer et al., 2015). Therefore, given Crus I and II's connections with well-known nodes of the cerebral “fronto-parietal attention network” it is not surprising that lesions in this region would impair covert attention.

Previous studies examining covert attention in cerebellar patients have noted that damage to regions of the cerebellum that are known to be involved in the control of eye movements also led to deficits in covert attention (Townsend et al., 1999; Baier et al., 2010). These findings were recently corroborated by a neuroimaging study in healthy individuals that observed significant BOLD activity in cerebellar lobule VI for both covert attention, as well as eye movements (Striemer et al., 2015). In the current study we did not observe any relationship between damage in lobule VI and deficits in covert attention. This is because we specifically *excluded* patients with gross oculomotor deficits during clinical examination in order to facilitate our search for regions of the cerebellum that might be involved in covert attention that are *not* traditionally thought to be involved in oculomotor control. However, it is important to note that some previous imaging and electrical stimulation studies have linked Crus I and II with eye movement control (see Ron and Robinson, 1973; Straube et al., 1997; Dieterich et al., 2000). Interestingly, there are links between previous work and the current findings which have highlighted the role of more lateral cerebellar structures. As mentioned previously, both Crus I and II are connected with the FEF and the PPC (Lynch et al., 1994; Voogd, 2003; Strick et al., 2009; Prevosto et al., 2010; Buckner et al., 2011; Glickstein et al., 2011). In addition, functional connectivity analyses using fMRI have demonstrated that activity in lobule VI of the cerebellum is also linked to activity within the FEF and the PPC (Buckner et al., 2011; Striemer et al., 2015). This suggests that the cerebellum helps to control attention through providing inputs from both lobule VI as well as more lateral (i.e., Crus I and II) sub-regions to the same cortical target zones which are known to play important roles in controlling both attention and eye movements.

Interestingly, recent work has demonstrated that the lateral cerebellum is involved in both saccadic monitoring (as measured by the anti-saccade task; Peterburs et al., 2015) as well as saccadic updating (as measured by the double-step saccade task; Peterburs et al., 2013a,b). One interesting question that arises from this is what relationship, if any, might exist between deficits in saccade monitoring and remapping, and deficits in reflexive covert attention. Anti-saccades (as compared to pro-saccades) require the participant to voluntarily execute a saccade in the direction *opposite* that of an imperative target. As such the generation of anti-saccades involves *voluntary* (i.e., endogenous) as opposed to reflexive attention (Alivisatos and Milner, 1989; Danckert et al., 1998; Bartolomeo et al., 2001). In addition, anti-saccades tend to generate increased activity within the frontal lobes and posterior parietal cortex, as well as the lateral cerebellum (for a meta-analysis see Jamadar et al., 2013). In contrast, previous imaging and lesion studies have linked cerebellar damage primarily with *reflexive* rather than voluntary attention (Townsend et al., 1999; Baier et al., 2010; Striemer et al., 2015). In terms of saccadic remapping, previous studies have noted that there are strong links between visual working memory and saccadic remapping (Vuilleumier et al., 2007; Vasquez and Danckert, 2008). Therefore, deficits in saccadic remapping following cerebellar damage may be more likely to be linked with problems in visual working memory or executive functions which are also known to rely on lateral cerebellar structures (for a review see Stoodley and Schmahmann, 2009b). Thus, it is unclear whether or not deficits in saccade monitoring and saccadic remapping are related to deficits in covert attention in patients with cerebellar lesions. This could be examined in future studies by measuring covert attention performance and saccade monitoring and remapping performance in the same patients.

One potential criticism of our work is that we did not use an eye tracker to monitor fixation in our participants. Although we did not directly monitor fixation throughout our experiment we do not feel as though this poses a problem for the interpretation of our results. First, both patients and controls performed a number of practice trials prior to the main experiment in which their eyes were directly monitored by the experimenter. None of the patients or controls had any trouble fixating during the practice trials. In addition, we specifically excluded cerebellar patients with gross oculomotor deficits which makes fixation problems much less likely in our patient group. Finally, differences in eye movements cannot explain the primary finding in our study which was a reduced cuing effect in cerebellar patients at the 50 ms SOA. Specifically, in trials with a 50 ms SOA the time between the cue and the target is too short for participants to execute an eye movement to the target location before the target appears. Therefore, the differences observed between patients and controls at the 50 ms SOA cannot be explained by eye movements.

Another potential criticism of our work concerns the small sample size (*n* = 11) we investigated in the current study. Part of the reason for our smaller sample size was the fact that we only included patients in our study who had a circumscribed cerebellar lesion or tumor, who did not have any gross motor or oculomotor deficits. Although a smaller sample does limit the interpretability of our lesion analysis to some degree, it should be noted that the number of patients we used to link specific cerebellar structures to covert attention is consistent with previous studies (Townsend et al., 1999; Baier et al., 2010). Specifically, Townsend et al. (1999) studied a group of 9 cerebellar patients, whereas Baier et al. (2010) studied a larger group of 26 cerebellar patients. Although Baier et al. (2010) studied a larger group of patients, they only observed covert attention deficits in a smaller sub-group of 8 patients. Thus, our anatomical conclusions are based a sample size that is similar to that used in previous studies.

Although lobule VI and vermal structures, as well as lateral portions of the cerebellum (i.e., Crus I and II) play a role in reflexive covert attention, it is not yet clear what specific role these structures play in controlling covert attention. What we can say for certain is that the cerebellar structures involved in covert attention (both midline and lateral regions) provide input to areas in the well-characterized fronto-parietal attention network (Buckner et al., 2011; Striemer et al., 2015) that act to control both attention and eye movements (Corbetta et al., 1998; Corbetta and Shulman, 2002).

## Conclusion

In conclusion, the results from the current study provide converging evidence that the cerebellum is critically involved in controlling the rapid deployment of reflexive covert attention. Furthermore, our results also provide important new insights into the role of the lateral cerebellar regions Crus I and II in covert attention.

### Conflict of interest statement

The authors declare that the research was conducted in the absence of any commercial or financial relationships that could be construed as a potential conflict of interest.

## Footnotes

^1^Although our initial correlation analysis revealed a trend toward a negative correlation between cuing effect and time post-injury this was driven by a single patient who was an outlier. Once this patient was removed the trend disappeared [*r*_(10)_ = −0.30, *p* = 0.41].
