# Supplementary material for: Deficits in reflexive covert attention following cerebellar injury
Source: Front Hum Neurosci. 2015 Aug 4;9:428. doi: 10.3389/fnhum.2015.00428 (PMC4523795; doi:10.3389/fnhum.2015.00428)
Supplement: Supplementary file 1 [file Image1.PDF]

Supplementary Figures 1-11. Each figure depicts the lesion for each individual patient (n=11). Lesioned areas in each patient were manually traced onto a series of 12-4mm thick axial cerebellar slices based on the templates first developed by Tatu and colleagues (Tatu et al., 1996). Structural labels and anatomical boundaries were determined by comparing sections of the cerebellar template with horizontal MRI and histology sections from the MRI Atlas of the Human Cerebellum (Jeremy D. Schmahmann, 2000). Slice 1 starts at the most inferior portion of the cerebellum and moves upward in 4mm increments towards more superior portions of the cerebellum. Gray portions represent the area of the patient's lesions. The lesion overlay for the overall group is presented in the main manuscript.

Patient 1

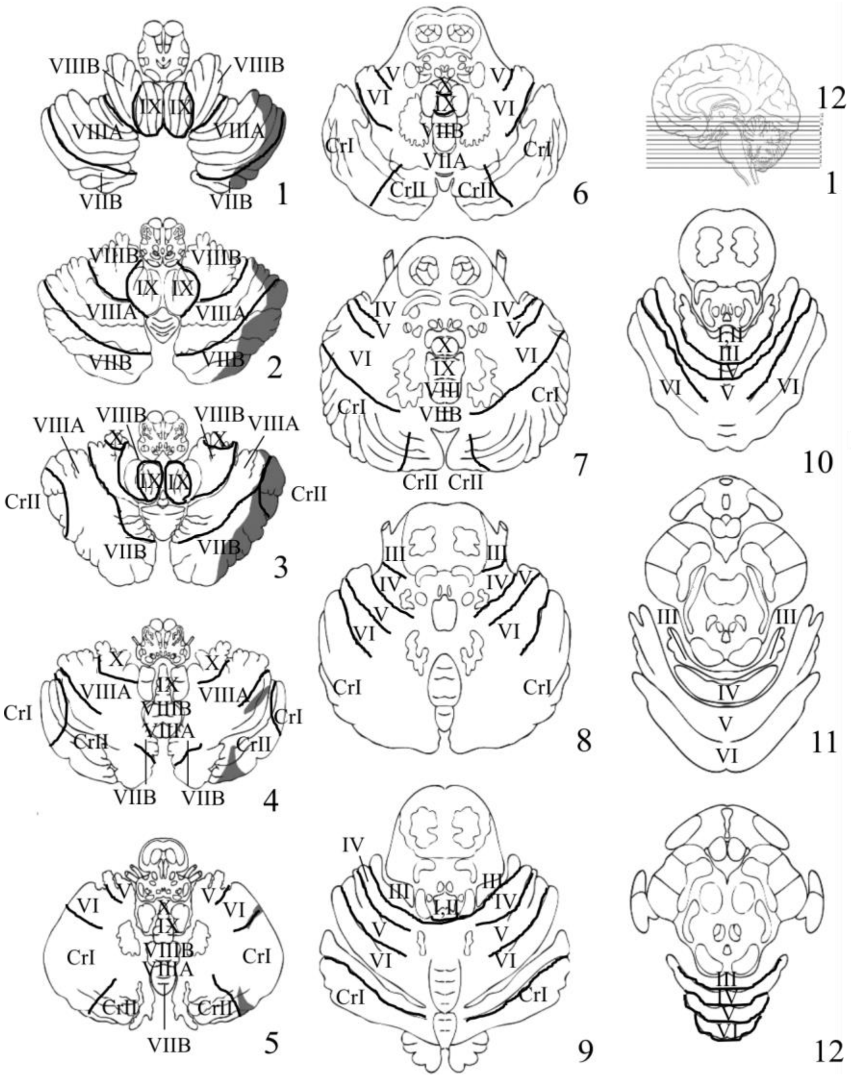
VIIIB
VIIIB
IX IX
VIIIA
VIIIA
VIIB
VIIB
1
V
V
VI
VI
X
IX
VIIB
CrI
CrI
VIIA
CrII
CrII
6
12
1
VIIIB
VIIIB
IX IX
VIIIA
VIIIA
VIIB
VIIB
2
IV
IV
V
V
VI
VI
X
IX
VIII
CrI
CrI
VIIB
CrII CrII
7
I,II
III
IV
V
VI
VI
10
VIIIA
VIIIB
VIIIB
VIIIA
X
X
IX IX
CrII
CrII
VIIB
VIIB
3
III
III
IV
IV
V
V
VI
VI
CrI
CrI
8
III
III
IV
V
VI
11
X
X
IX
VIIIA
VIIIA
VIIIB
VIIIA
CrI
CrI
CrII
CrII
VIIB
VIIB
4
IV
III
III
I,II
IV
V
V
VI
VI
CrI
CrI
9
VI
V
V
VI
X
IX
VIIIB
VIIIA
CrI
CrI
CrII
CrII
VIIB
5
III
IV
V
VI
12

Patient 2

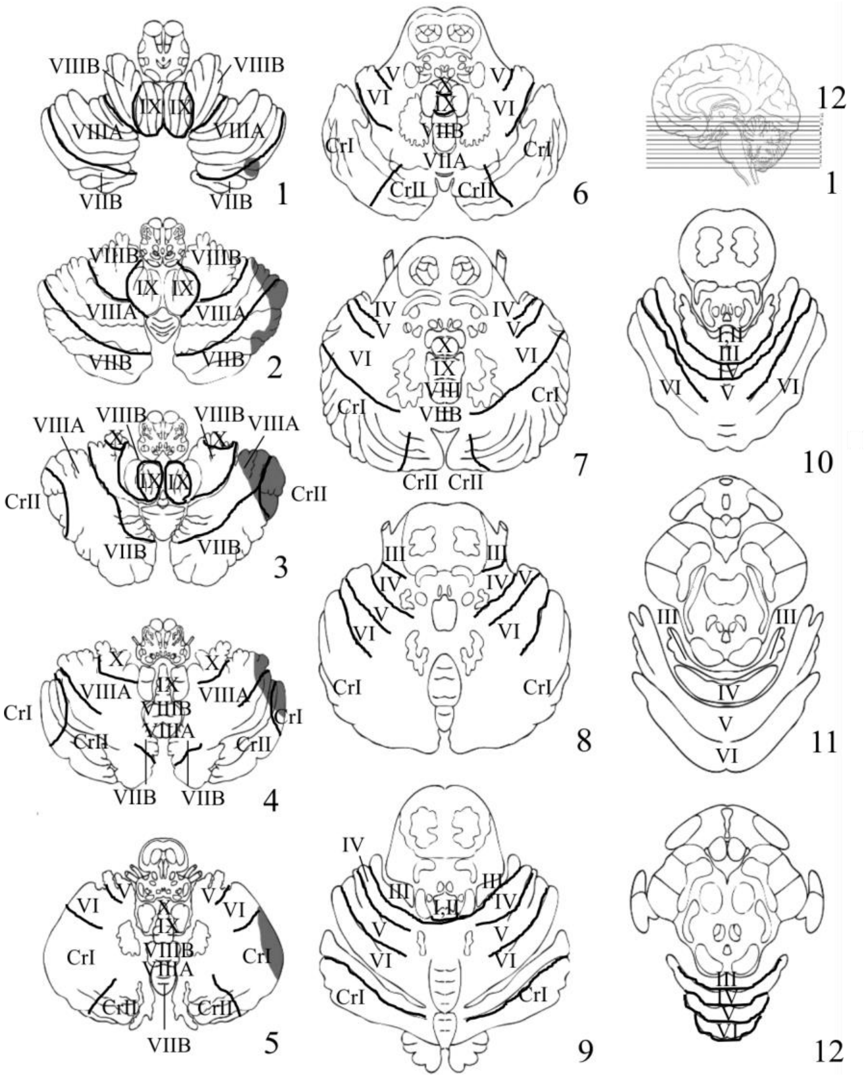
VIIIB
VIIIB
IX
IX
VIIIA
VIIIA
VIIB
VIIB
1
VIIIB
VIIIB
IX
IX
VIIIA
VIIIA
VIIB
VIIB
2
VIIIA
VIIIB
VIIIB
VIIIA
X
X
IX
IX
CrII
CrII
VIIB
VIIB
3
X
X
IX
VIIIA
VIIIA
VIIIB
VIIIA
CrI
CrI
CrII
CrII
VIIB
VIIB
4
VI
V
V
VI
X
IX
VIIIB
VIIIA
CrI
CrI
CrII
CrII
VIIB
5
V
V
VI
VI
X
IX
VIIB
VIIA
CrI
CrI
CrII
CrII
6
IV
IV
V
V
X
VI
VI
IX
VIII
CrI
CrI
VIIB
CrII
CrII
7
III
III
IV
IV
V
V
VI
VI
CrI
CrI
8
IV
III
III
I-II
IV
V
V
VI
VI
CrI
CrI
9
12
1
I-II
III
IV
VI
V
VI
10
III
III
IV
V
VI
11
III
IV
V
VI
12

Patient 3

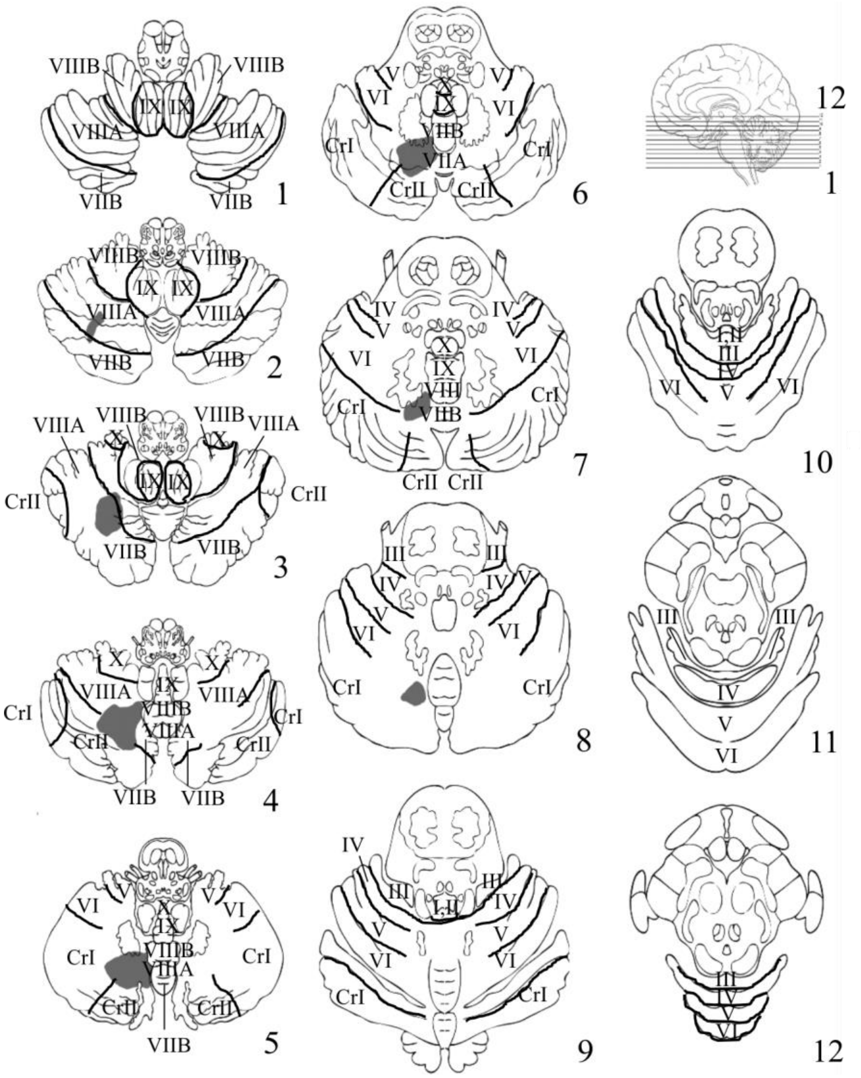
VIIIB
VIIIB
IX IX
VIIIA
VIIIA
VIIB
VIIB
1
VIIIB
VIIIB
IX IX
VIIIA
VIIIA
VIIB
VIIB
2
VIIIA
VIIIB
VIIIB
VIIIA
X
X
IX IX
CrII
CrII
VIIB
VIIB
3
X
X
IX
VIIIA
VIIIA
VIIIB
CrI
CrI
CrII
VIIIA
CrII
VIIB
VIIB
4
VI
V
V
VI
X
IX
VIIIB
CrI
CrI
VIIIA
CrII
CrII
VIIB
5
V
V
VI
X
IX
VI
VIIIB
CrI
CrI
VIIA
CrII
CrII
6
IV
IV
V
V
X
VI
VI
IX
VIII
CrI
CrI
VIIB
CrII
CrII
7
III
III
IV
IV
V
V
VI
VI
CrI
CrI
8
IV
III
III
I,II
IV
V
V
VI
VI
CrI
CrI
9
12
1
III
IV
VI
V
VI
10
III
III
IV
V
VI
11
III
IV
V
VI
12

Patient 4

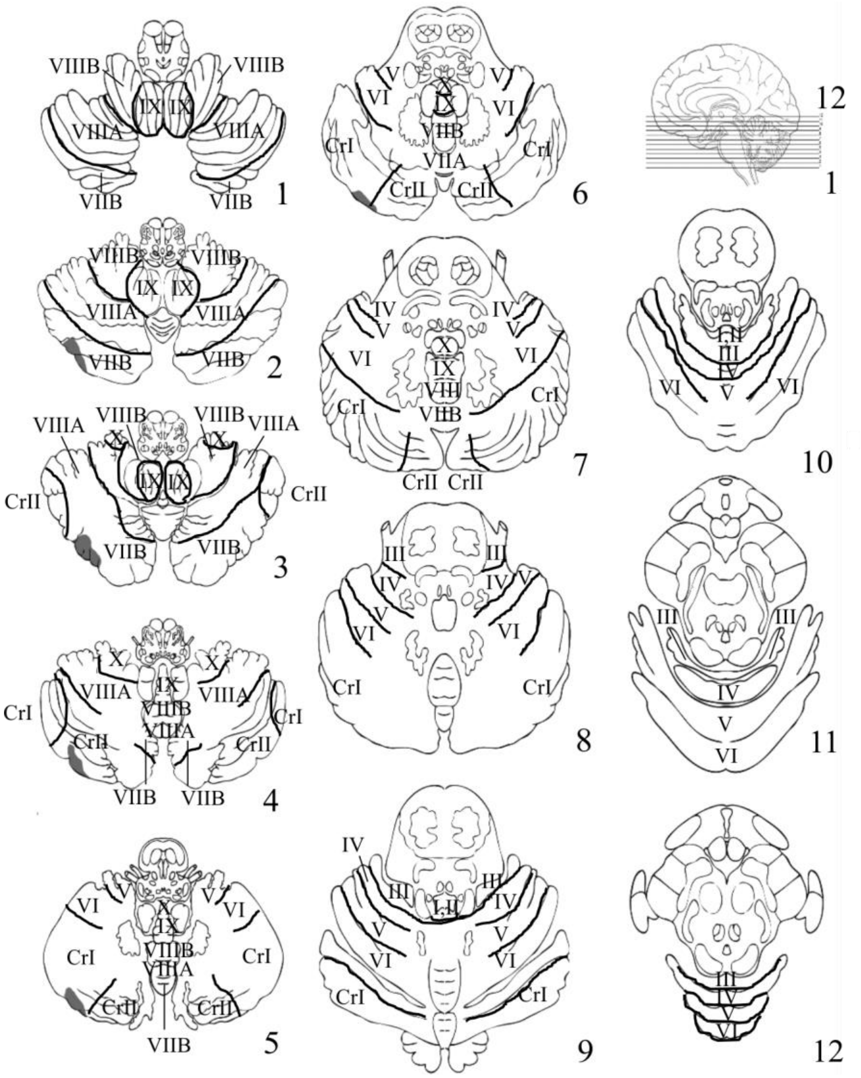

VIIIB
VIIIB
IX
IX
VIIIA
VIIIA
VIIB
VIIB
1
V
V
X
IX
VI
VI
VIIIB
CrI
CrI
VIIA
CrII
CrII
6
12
1
VIIIB
VIIIB
IX
IX
VIIIA
VIIIA
VIIB
VIIB
2
IV
IV
V
V
X
VI
IX
VI
VIII
CrI
CrI
VIIB
CrII
CrII
7
III
IV
VI
V
VI
10
VIIIA
VIIIB
VIIIB
VIIIA
X
X
CrII
CrII
IX
IX
VIIB
VIIB
3
III
III
IV
IV
V
V
VI
VI
CrI
CrI
8
III
III
IV
V
VI
11
X
X
IX
VIIIA
VIIIA
CrI
VIIIB
CrI
CrII
CrII
VIIB
VIIB
4
V
V
VI
VI
X
IX
CrI
CrI
VIIIB
VIIIA
CrII
CrII
VIIB
5
IV
III
III
IV
I, II
V
V
VI
VI
CrI
CrI
9
III
IV
V
VI
12

Patient 5

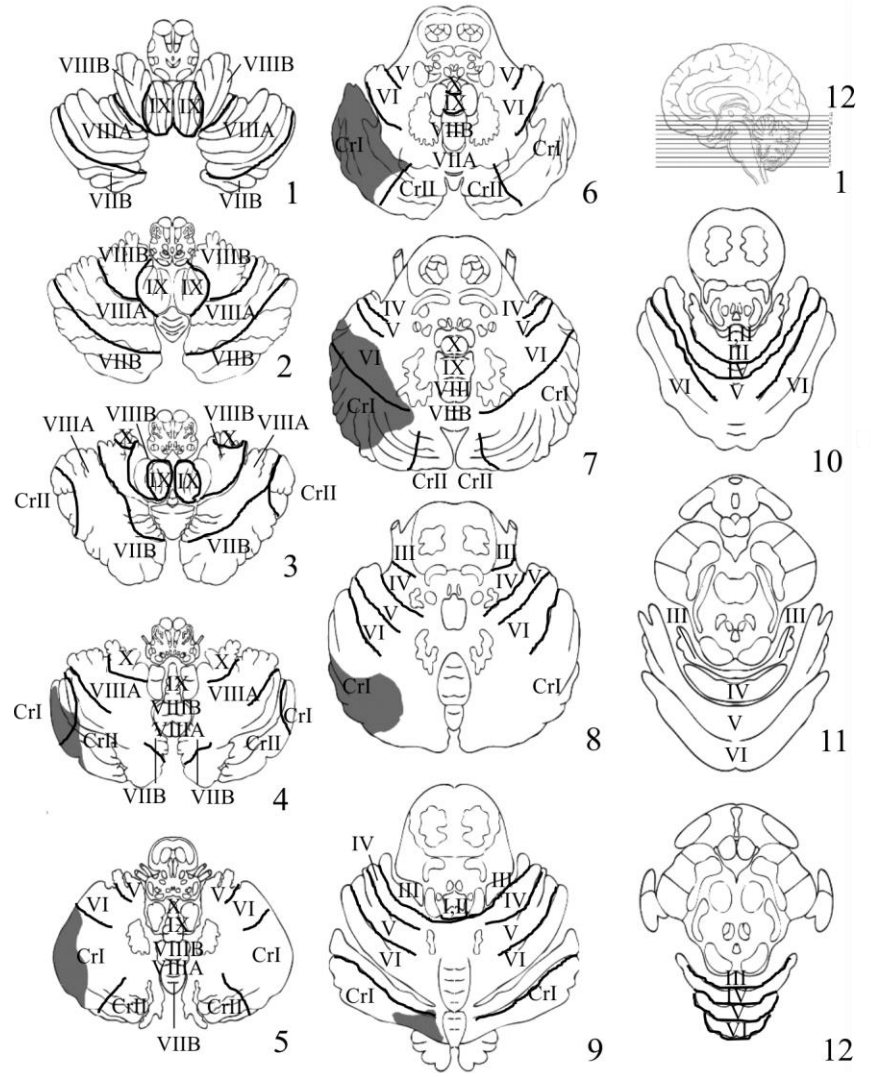

VIIIB
VIIIB
IX IX
VIIIA
VIIIA
VIIB
VIIB
1
V
V
VI
X
IX
VI
VIIB
CrI
CrI
VIIA
CrII
CrII
6
12
1
VIIIB
VIIIB
IX IX
VIIIA
VIIIA
VIIB
VIIB
2
IV
IV
V
V
X
VI
VI
IX
VIII
CrI
CrI
VIIB
CrII CrII
7
I,II
III
IV
V
VI
VI
10
VIIIA
VIIIB
VIIIB
VIIIA
X
X
CrII
IX IX
CrII
VIIB
VIIB
3
III
III
IV
IV
V
V
VI
VI
CrI
CrI
8
III
III
IV
V
VI
11
X
X
IX
VIIIA
VIIIA
CrI
VIIIB
CrI
VIIIA
CrII
CrII
VIIB
VIIB
4
IV
III
III
IV
I,II
V
V
VI
VI
CrI
CrI
9
VI
V
V
VI
X
IX
VIIIB
CrI
CrI
VIIIA
CrII
CrII
VIIB
5
III
IV
V
VI
12

Patient 6

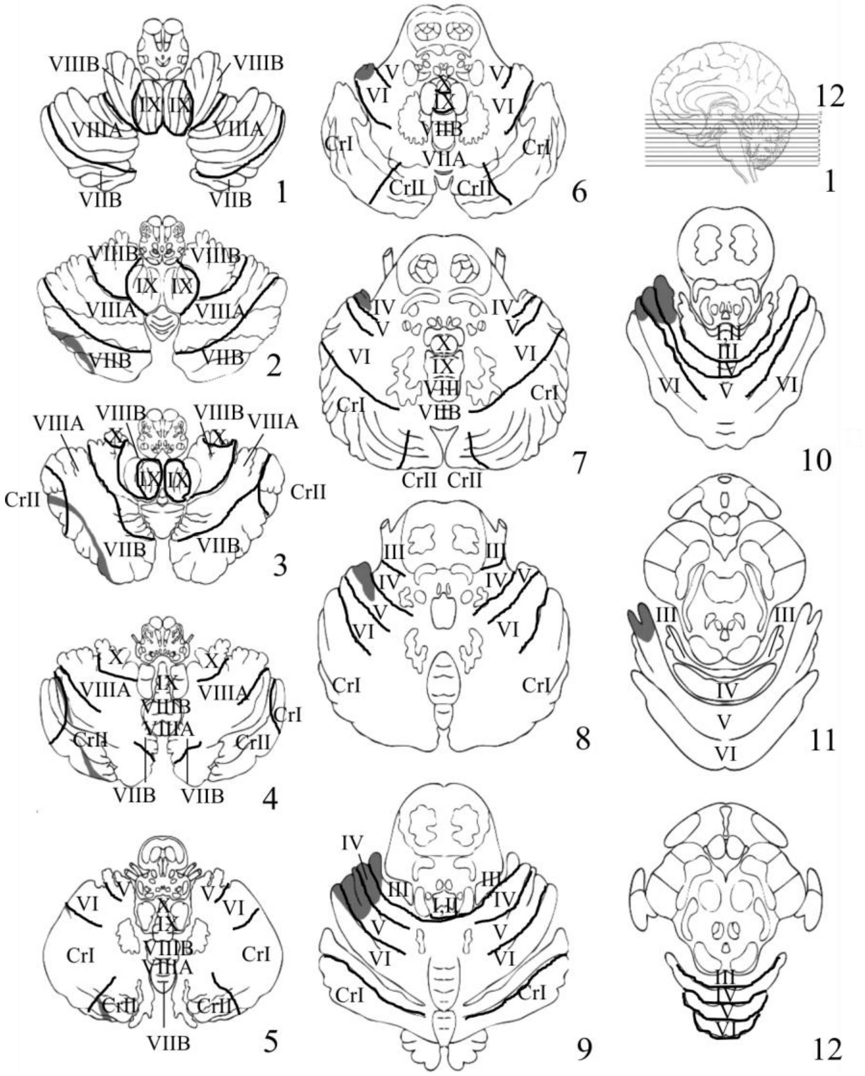

VIIIB
VIIIB
IX IX
VIIIA
VIIIA
VIIB
VIIB
1
V
V
VI
X
IX
VI
VIIIB
CrI
CrI
VIIA
CrII
CrII
6
12
1
VIIIB
VIIIB
IX
IX
VIIIA
VIIIA
VIIB
VIIB
2
IV
IV
V
V
VI
X
IX
VI
VIII
CrI
CrI
VIIB
CrII
CrII
7
I,II
III
IV
V
VI
VI
10
VIIIA
VIIIB
VIIIB
VIIIA
X
X
IX IX
CrII
CrII
VIIB
VIIB
3
III
III
IV
IV
V
V
VI
VI
CrI
CrI
8
III
III
IV
V
VI
11
X
X
IX
VIIIA
VIIIB
VIIIA
CrI
VIIIA
CrII
CrII
VIIB
VIIB
4
VI
V
V
VI
X
IX
CrI
VIIIB
CrI
VIIIA
CrII
CrII
VIIB
5
IV
III
III
I,II
IV
V
V
VI
VI
CrI
CrI
9
III
IV
V
VI
12

Patient 7

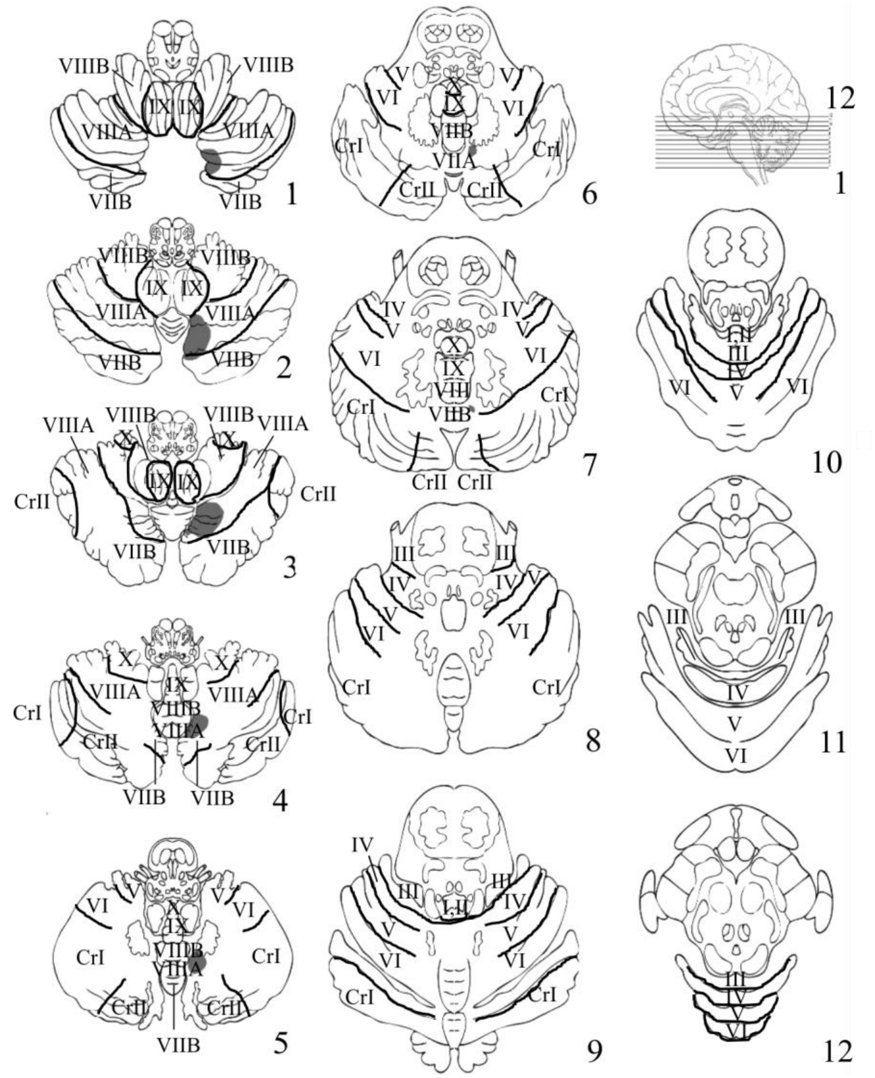

VIIIB
VIIIB
IX
IX
VIIIA
VIIIA
VIIB
VIIB
1
VIIIB
VIIIB
IX
IX
VIIIA
VIIIA
VIIB
VIIB
2
VIIIA
VIIIB
VIIIB
VIIIA
X
X
IX
IX
CrII
CrII
VIIB
VIIB
3
X
X
IX
VIIIA
VIIIA
VIIIB
VIIIA
CrI
CrI
CrII
CrII
VIIB
VIIB
4
VI
V
V
VI
X
IX
VIIIB
VIIIA
CrI
CrI
CrII
CrII
VIIB
5
V
V
VI
X
IX
VI
VIIB
CrI
VIIA
CrI
CrII
CrII
6
IV
IV
V
V
X
VI
VI
IX
VIII
CrI
CrI
VIIB
CrII
CrII
7
III
III
IV
IV
V
V
VI
VI
CrI
CrI
8
IV
III
III
I-II
IV
V
V
VI
VI
CrI
CrI
9
12
1
I-II
III
IV
VI
V
VI
10
III
III
IV
V
VI
11
III
IV
V
VI
12

Patient 8

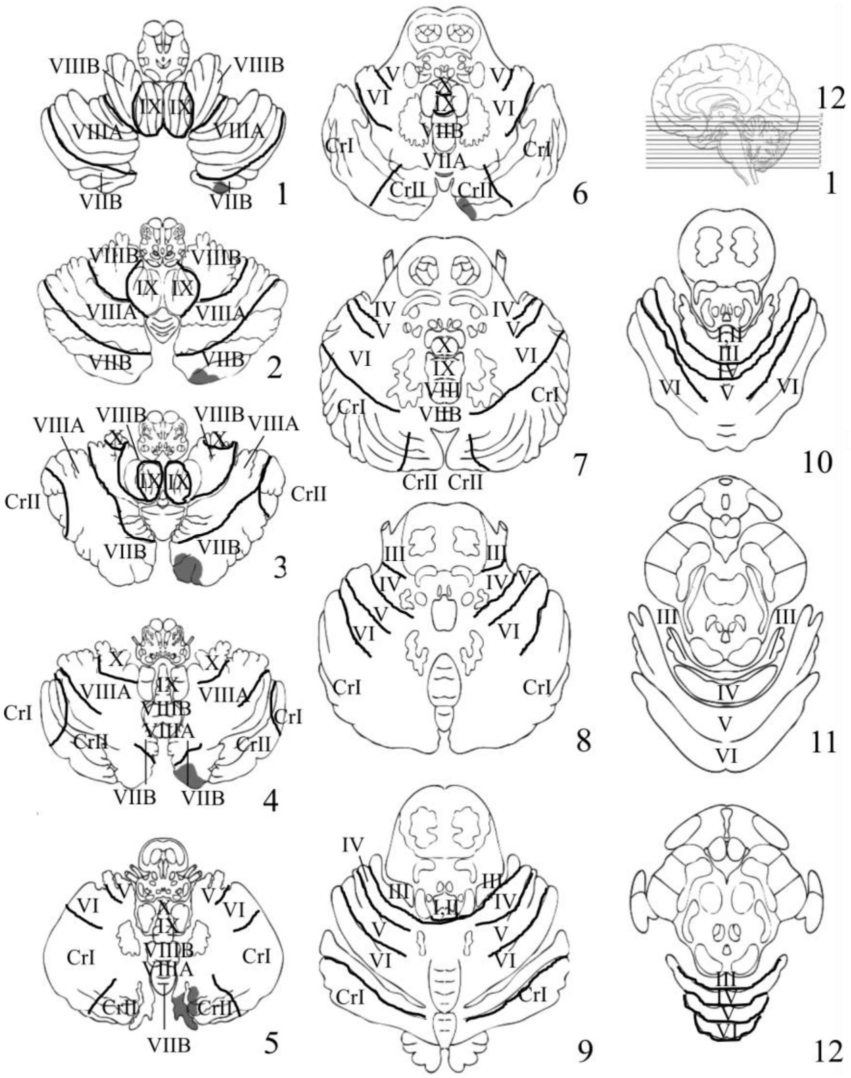

VIIIB
VIIIB
IX
IX
VIIIA
VIIIA
VIIB
VIIB
1
VIIIB
VIIIB
IX
IX
VIIIA
VIIIA
VIIB
VIIB
2
VIIIA
VIIIB
VIIIB
VIIIA
X
X
IX
IX
CrII
CrII
VIIB
VIIB
3
X
X
IX
VIIIA
VIIIA
VIIIB
VIIIA
CrI
CrI
CrII
CrII
VIIB
VIIB
4
VI
V
X
V
VI
IX
VIIIB
VIIIA
CrI
CrI
CrII
CrII
VIIB
5
V
V
VI
X
IX
VI
VIIIB
CrI
CrI
VIIA
CrII
CrII
6
IV
IV
V
V
X
VI
VI
IX
VIII
CrI
CrI
VIIB
CrII
CrII
7
III
III
IV
IV
V
V
VI
VI
CrI
CrI
8
IV
III
III
I,II
IV
V
V
VI
VI
CrI
CrI
9
12
1
I,II
III
IV
VI
V
VI
10
III
III
IV
V
VI
11
III
IV
V
VI
12

Patient 9

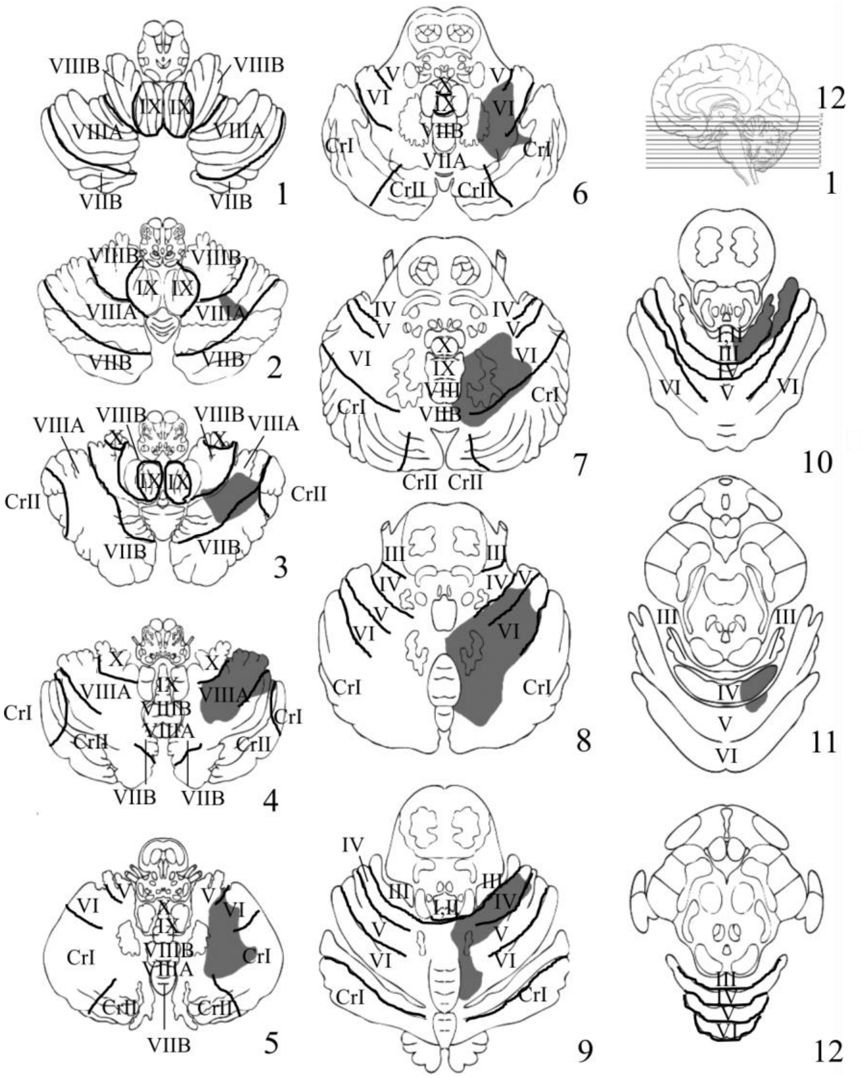

Patient 10

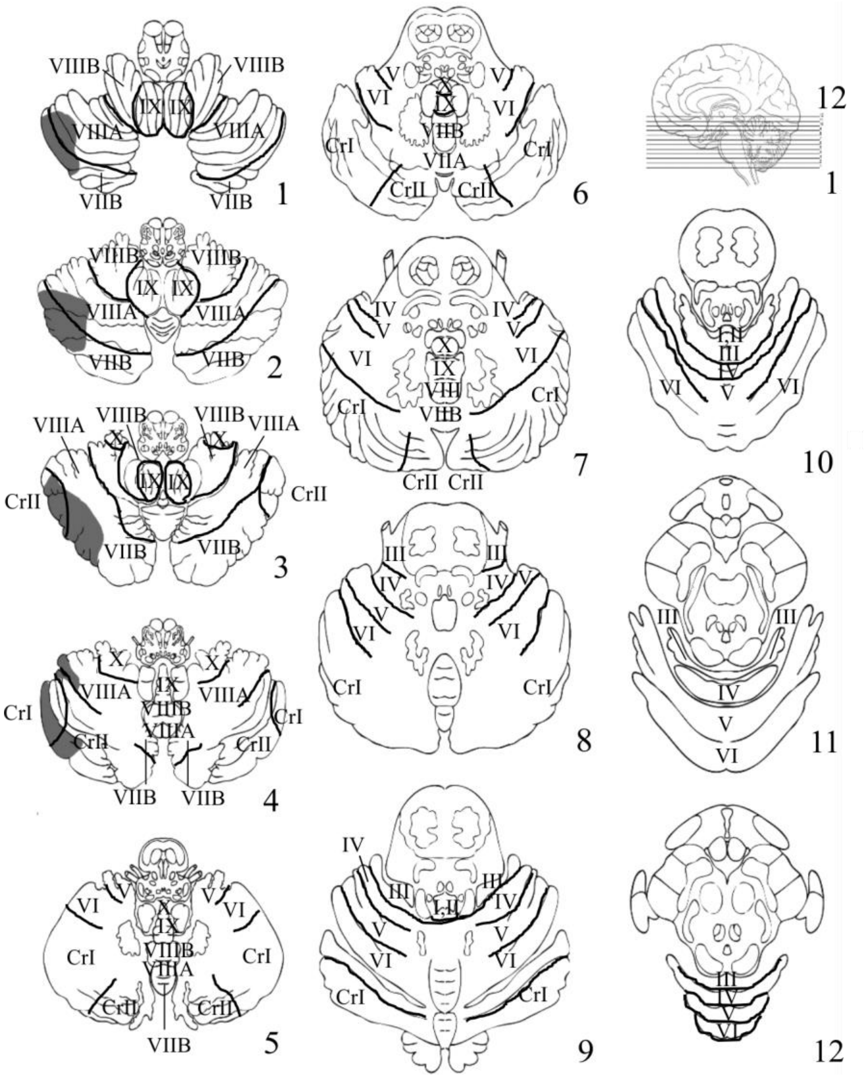

VIIIB
VIIIB
IX
IX
VIIIA
VIIIA
VIIB
VIIB
1
V
V
VI
VI
X
IX
VIIIB
CrI
CrI
VIIA
CrII
CrII
6
12
1
VIIIB
VIIIB
IX
IX
VIIIA
VIIIA
VIIB
VIIB
2
IV
IV
V
V
X
VI
VI
IX
VIII
CrI
CrI
VIIB
CrII
CrII
7
III
III
IV
VI
V
VI
10
VIIIA
VIIIB
VIIIB
VIIIA
X
X
IX
IX
CrII
CrII
VIIB
VIIB
3
III
III
IV
IV
V
V
VI
VI
CrI
CrI
8
III
III
IV
V
VI
11
X
X
IX
VIIIA
VIIIA
VIIIB
CrI
CrI
VIIIA
CrII
CrII
VIIB
VIIB
4
VI
V
V
VI
X
IX
CrI
VIIIB
CrI
VIIIA
CrII
CrII
VIIB
5
IV
III
III
I,II
IV
V
V
VI
VI
CrI
CrI
9
III
IV
V
VI
12

Patient 11

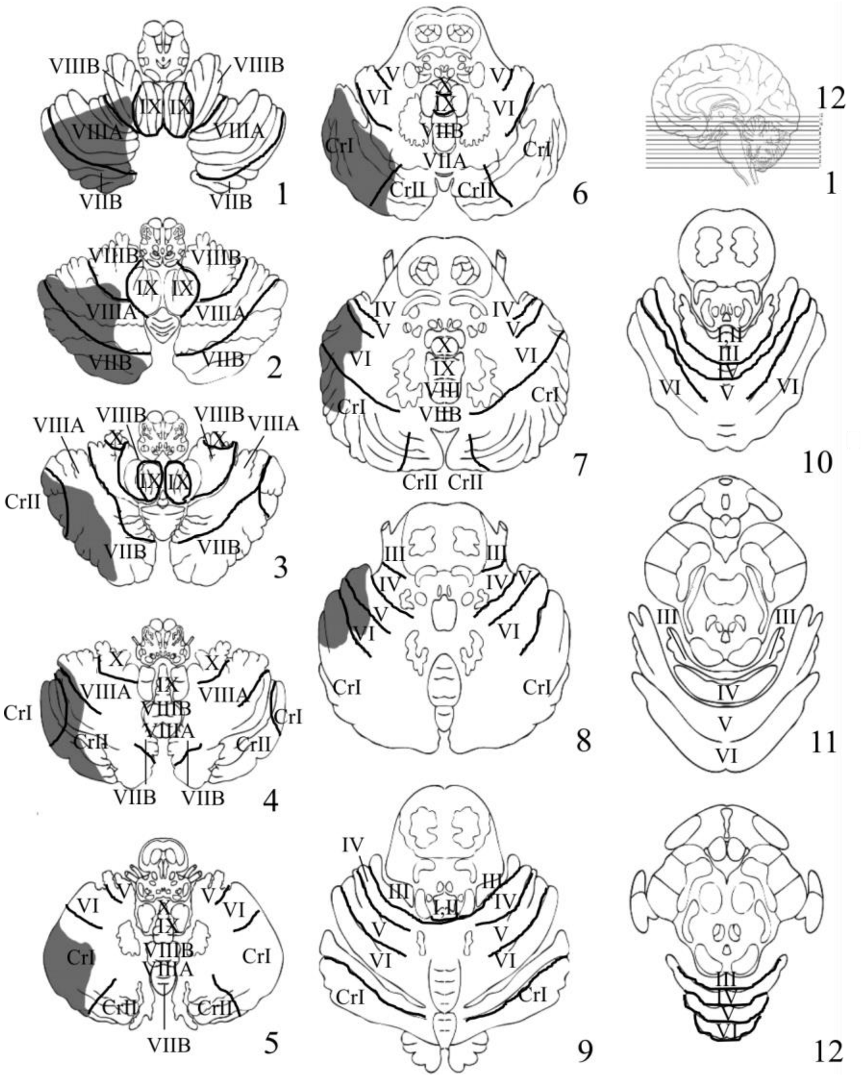
VIIIB
VIIIB
IX IX
VIIIA
VIIIA
VIIB
VIIB
1
V
V
VI
X
IX
VI
VIIB
CrI
CrI
VIIA
CrII
CrII
6
12
1
VIIIB
VIIIB
IX IX
VIIIA
VIIIA
VIIB
VIIB
2
IV
IV
V
V
X
VI
IX
VI
VIII
CrI
CrI
VIIB
CrII CrII
7
VI
V
VI
10
VIIIA
VIIIB
VIIIB
VIIIA
X
X
IX IX
CrII
VIIB
VIIB
3
III
III
IV
IV
V
V
VI
VI
CrI
CrI
8
III
III
IV
V
VI
11
X
X
IX
VIIIA
VIIIA
VIIIB
CrI
CrI
CrII
VIIIA
CrII
VIIB
VIIB
4
VI
V
V
VI
X
IX
CrI
VIIIB
CrI
VIIIA
CrII
CrII
VIIB
5
IV
III
III
IV
V
V
VI
VI
CrI
CrI
9
III
12
